# Generative adversarial network based adaptive data augmentation for handwritten Arabic text recognition

**DOI:** 10.7717/peerj-cs.861

**Published:** 2022-01-25

**Authors:** Mohamed Eltay, Abdelmalek Zidouri, Irfan Ahmad, Yousef Elarian

**Affiliations:** 1 Electrical Engineering Department, King Fahd University of Petroleum & Minerals, Dhahran, Saudi Arabia; 2 IRC for Intelligent Secure Systems, King Fahd University of Petroleum and Minerals, Dhahran, Saudi Arabia; 3 Information and Computer Science Department, King Fahd University of Petroleum & Minerals, Dhahran, Saudi Arabia; 4 Cambrian College, Sudbury, Ontario, Canada

**Keywords:** Adaptive data augmentation, Deep learning neural Networks, Arabic handwriting recognition, Handwritten text generation, Generative adversarial networks, Convolutional neural networks

## Abstract

Training deep learning based handwritten text recognition systems needs a lot of data in terms of text images and their corresponding annotations. One way to deal with this issue is to use data augmentation techniques to increase the amount of training data. Generative Adversarial Networks (GANs) based data augmentation techniques are popular in literature especially in tasks related to images. However, specific challenges need to be addressed in order to effectively use GANs for data augmentation in the domain of text recognition. Text data is inherently imbalanced in terms of frequency of different characters appearing in training samples and the training data as a whole. GANs trained on the imbalanced dataset leads to augmented data that does not represent the minority characters well. In this paper, we present an adaptive data augmentation technique using GANs that deals with the issue of class imbalance arising in text recognition problems. We show, using experimental evaluations on two publicly available datasets for handwritten Arabic text recognition, that the GANs trained using the presented technique is effective in dealing with class imbalanced problem by generating augmented data that is balanced in terms of character frequencies. The resulting text recognition systems trained on the balanced augmented data improves the text recognition accuracy as compared to the systems trained using standard techniques.

## Introduction

Handwritten text recognition is a classical problem in the field of computer vision and pattern recognition (Altwaijry & Al-Turaiki, 2021). Major fields of automation such as vehicle plate recognition and digital postal services rely on the development of text recognition. Comprehensive handwriting datasets are required to train and test text recognition systems. As a result, researchers are now working to improve the effectiveness and quality of text recognition (Elarian et al., 2015) by using synthesized data to expand the training sets.

One of the important researched problems in this field is the synthesis of personal handwriting. Handwriting synthesis is the process by which a computer generates data that resembles human handwriting. It aims to create handwritten image samples in the same style as the target writer. This is especially useful for training handwriting recognition systems. It can be seen as a reverse process of handwriting recognition because it converts input text into image samples, whereas recognition maps handwritten image samples into digital text. Handwriting synthesis has grown rapidly as a result of its applications, such as improving text recognition systems (in terms of overall performance, stability, and speed), font customization, CAPTCHAS for distinguishing a human from a computer, and forgery detection (Elarian et al., 2014).

In recent years, Generative Adversarial Networks (GANs) have proven to be extremely successful in a wide range of image processing applications (Antoniou, Storkey & Edwards, 2017; Antipov, Baccouche & Dugelay, 2017). GANs can now be used to generate photo-realistic images of objects such as human faces, animals, and indoor or outdoor scenes. In addition, GANs can be used to translate images from one domain to another, generate high-definition images from low-definition images, and so on (Creswell et al., 2018).

To the best of our knowledge, only a few works have been published on Arabic text image synthesis using generative adversarial networks. We present an Arabic handwriting synthesis system where we generate the necessary text to balance the dataset using an adaptive data augmentation method. Then, we train a generative adversarial network based words-synthesizer to produce images of Arabic words that appear to be handwritten by humans. We tested our work using a state-of-the-art deep learning based handwriting recognition system, and the results show that the presented method improves the recognition accuracy.

The following contributions are made in this work: we review adversarial architectures that generate realistic handwritten texts. Then, we present a modified adversarial architecture that can improve handwritten Arabic text synthesis by generating augmented training dataset which is balanced in terms of the distribution of the characters of the Arabic script. Finally, we evaluate handwriting text recognition systems using the training sets augmented with GAN generated text images and report the results on two publicly available datasets.

The rest of the paper is organized as follows: In ‘Literature Review’, we present a summary of the related works. In ‘Introduction to Arabic handwriting’, we present a brief introduction to Arabic script and their peculiarities. Approaches to generate handwritten Arabic text images is presented in ‘Approaches to generate handwritten arabic words’ with particular emphasis on GANs for text image synthesis. Our methodology for data augmentation using GANs is presented in ‘A two-stage generative adversarial network based adaptive data augmentation technique’. In ‘Experimentation and results’, we present the experiments and discuss the results. Finally, conclusions are presented in ‘Conclusion’.

## Literature Review

Neural networks and deep learning have aided research in handwriting synthesis. Traditional machine learning algorithms were used by the researchers to build strokes and model characters, but none of these methods produced good human-like handwriting (Alonso, Moysset & Messina, 2019; Jha & Cecotti, 2020). Most of these findings were made prior to the advancement of neural networks. Graves (2013) demonstrated that by predicting one data point at a time, Recurrent Neural Networks (RNN) with Long Short-Term Memory (LSTM) can generate complex sequences with long-range structure. The method was tested using text which contained discrete data and online handwriting where the data are time-stamped. It was then extended to handwriting synthesis by allowing the network to make predictions based on the sequence of characters in a text. This resulted in a system capable of producing highly realistic cursive handwriting in a variety of styles.

Saabni & El-Sana (2013) presented an efficient system that used multiple appearances of each Arabic character to generate prototypes for each word in a lexicon. For each character in each position, large sets of different shapes were created. The valid shapes for each word-part are then generated using these sets. A large number of valid permutations for each word makes practical training and searching for tasks like script recognition and word spotting impossible. Authors used dimensionality reduction and clustering techniques to keep these datasets tractable while maintaining their ability to represent a wide range of handwriting styles. A dataset for offline script recognition was also created from the online strokes using a standard dilation technique, with special attention paid to mimicking the pen’s path. Authors also looked at and tried out a few different layout techniques for making words out of the generated word parts.

Elarian et al. (2015) presented an Arabic handwriting synthesis system that concatenates Arabic glyphs into words. To synthesize Arabic words, they used two concatenation models: the Extended-Glyph connection and the Synthetic-Extension connection. The system then injected the synthesized handwriting into a larger dataset to significantly improve recognition performance.

Ahmad & Fink (2015b) have investigated various handwritten Arabic text recognition approaches that did not rely on handwritten training sets by taking advantage of computer-generated text in different typefaces, unsupervised adaptation and recognition hypothesis on test sets to be used as training data.

Wigington et al. (2017) created two data augmentation and normalization techniques for Latin and French that, when combined with a CNN-LSTM, significantly improved recognition rates. They started by applying a novel method for normalizing profile images to both word and line images. In order to augment existing text images, they used random perturbations on a regular grid.

Alonso, Moysset & Messina (2019) presented a system for producing synthetic images of handwritten Arabic and French words based on Generative Adversarial Networks (GANs). They generated an embedding of the rendered word using bidirectional LSTM recurrent layers which was then fed into the generator network. They also modified the standard GAN by adding a text recognition auxiliary network. The system was then trained with a balanced adversarial and Connectionist Temporal Classification (CTC) loss. By combining these GAN extensions, users gained control over the generated word images’ textual content, resulting in images that appear to be realistic in both French and Arabic.

Jha & Cecotti (2020) presented a data augmentation approach for handwritten digit recognition using generative adversarial networks. The approach was tested on handwritten Latin, Bangla, Devanagari, and Oriya digits.

Fogel et al. (2020) have presented ScrabbleGAN, a semi-supervised method for producing handwritten Latin and French text images in a variety of styles and lexicons. ScrabbleGAN was developed using a novel generative model capable of producing word images of any length. Using their approach in semi-supervised mode, they demonstrated a performance boost over current supervised handwritten text recognition. Furthermore, their generator has complete control over the resulting text style. This allows them to change things like the cursive style of the text and the thickness of the pen stroke.

To improve class diversity, Eltay, Zidouri & Ahmad (2020) implemented an adaptive data augmentation approach. Each word in the dataset lexicon is given a weight by the algorithm. The weight is determined by the probability of each character occurring in a word such that words consisting of less frequent characters get higher probabilities.

## Introduction to arabic handwriting

Arabic is a Semitic language that has been used for thousands of years. It is the *lingua franca* for more than 20 countries. Arabic is considered one of the world’s top six major languages (Pereltsvaig, 2020). Arabic is written from right to left. Depending on its position in the word, each character can have up to four distinct shapes, in both handwriting and printed form. Table 1 shows the Arabic letters in their stand-alone shapes.

**Table 1:** Arabic letters in their stand-alone shapes.

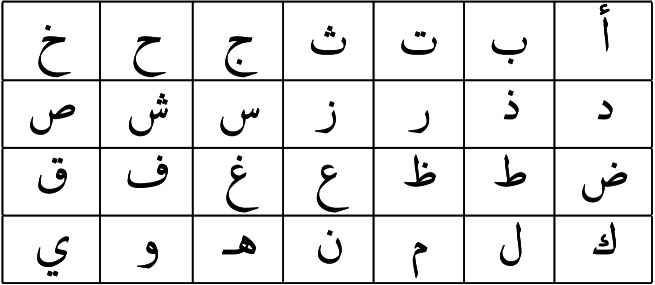

Handwritten Arabic text recognition presents unique challenges when compared to Latin script. These difficulties stem in part from the cursive nature of the script, the possibility of diacritics and diagonal strokes, the fact that characters change shapes depending on their location within the word, and the possibility of inter-word spaces. Arabic script is predominantly cursive, which means that the majority of the characters in a word are connected through a hypothetical horizontal line referred as a baseline. The Arabic language also has a large number of sub-words and characters which may be written with a variety of different characters or diacritics for each word. A few characters cannot link to their successors within words, and hence, their occurrence before the end of the word divides it into sub-words.

**Figure 1:**
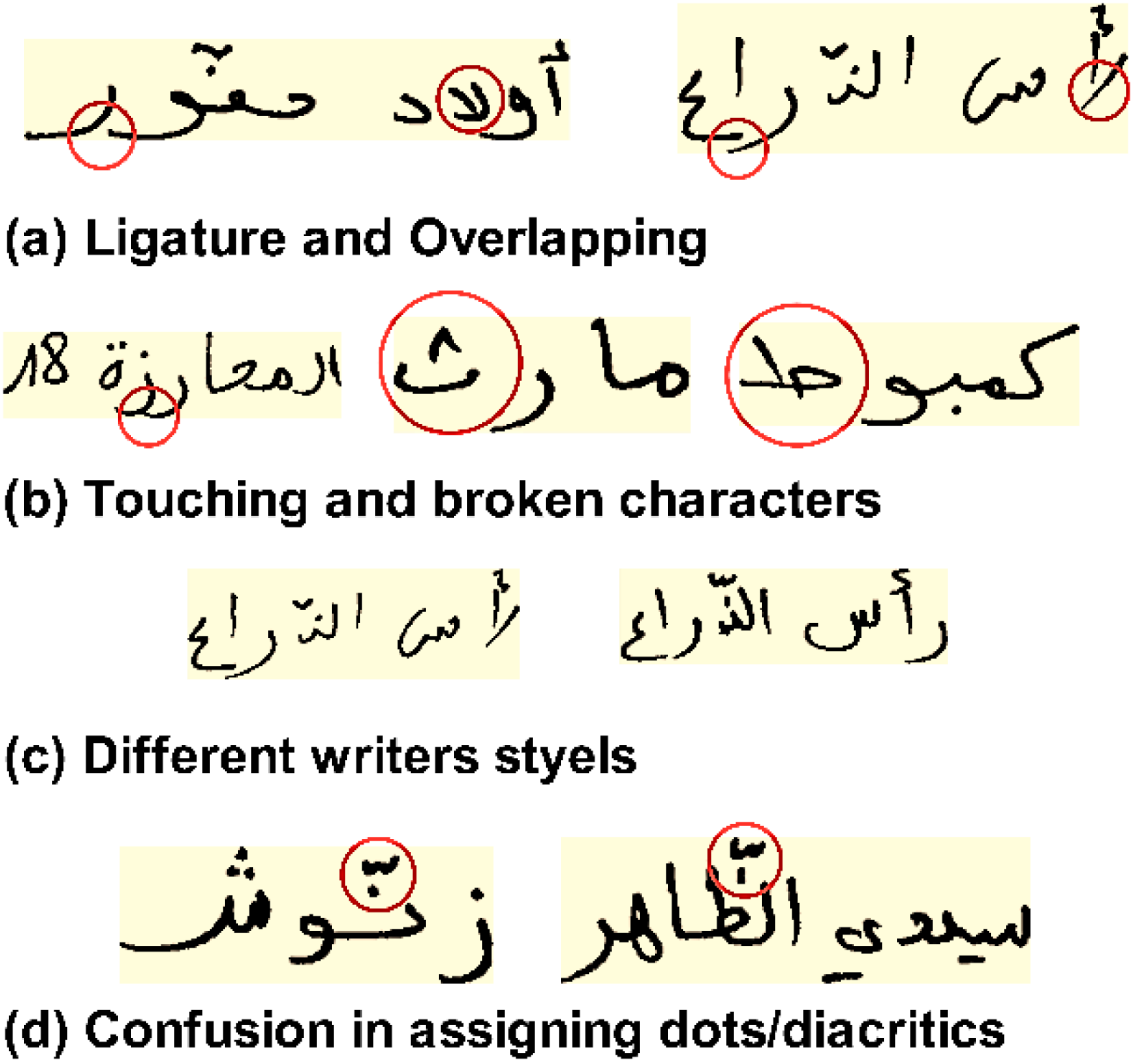
The most common difficulties encountered in handwriting Arabic recognition (A–D).

There is a lot of emphasis on dots in Arabic script. Although the character main bodies are similar, some are distinct due to the inclusion or exclusion of dots. For instance, the three characters, (Khaa 
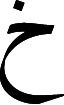
 ), (Haa 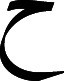 ), and (Jeem 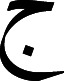 ) have similar main bodies and only differ by presence and location of a dot. Furthermore, diacritical imprints are used in Arabic to govern the articulation of words. These are extremely uncommon to encounter in handwritten texts. They are used frequently in educational documents and in contexts where ambiguity needs to be resolved. It is also possible to create ligatures by vertically consolidating different characters. In addition, each writer has a distinct handwriting style. Figure 1 shows the most common challenges and difficulties faced when dealing with handwritten Arabic text.

## Approaches to generate handwritten arabic words

Thousands of parameters need to be trained in even the smallest deep learning networks. There is a significant risk of over-fitting when using deep networks or when working with a small number of training images. Data augmentation, which enlarges the dataset artificially, is a common solution for reducing overfitting. On gray-scale images, affine transformations are the most common augmentation technique (Shorten & Khoshgoftaar, 2019). One way to augment training images is to use an image synthesis technique based on the GANs in order to enrich the training data. The method we propose here consists of two steps: In the first step, we use an adaptive data augmentation method, which we had previously described in Eltay, Zidouri & Ahmad (2020), to create a balanced dataset. Then, the expanded dataset will be used to train a GAN-based words-synthesizer to generate unique handwritten image words, thereby increasing class diversity.

### Adaptive data augmentation algorithm

Poor predictive performance can result from imbalanced data, especially for minority classes. Data augmentation is frequently used in a variety of deep learning approaches when only a small number of training samples are available. The original dataset’s class distribution is unaffected by rotation, position shifting, zooming, shearing, and other augmentation techniques. That is, if we have skewed data, it will remain skewed even after data augmentation. Any language or dataset can be affected by the problem of class imbalance based on differences in character frequencies. Observations like this are common when it comes to natural language texts (Wikipedia, 2021; Intellaren, 2021).

The adaptive data augmentation algorithm (Eltay, Zidouri & Ahmad, 2020) promotes more class-balance. This algorithm assigns a numerical weight to each word in the lexicon. The weight is calculated by taking the average probability of each class into account. Words containing less frequent characters will be augmented more than words containing characters appearing more frequently in texts. Table 2 shows the pseudo-code for our data-augmentation technique.

**Table 2:** Adaptive data augmentation algorithm.

Line:	dataset *db* with size *S*_*w*_ as the number of words and *S*_*u*_ as the total number of the characters in *db*
1.0:	Compute lexicon (size) *L* as the total number of distinct words in the input dataset *db*
2.0:	Let *N* be the total number of unique modeling units for *db*
3.0:	Let *F* be a fraction of *S*_*w*_ representing the total number of words that we need to augment to *db*
4.0:	For each Unit *n*
4.1:	Compute probability *p*_*n*_ as: (count of *n*)/*S*_*u*_
5.0:	For each word *W*_*i*_ in (*L*) the lexicon
5.1:	Compute the length of the word *l* as the number of units it contains
5.2:	Compute a weight as ${\mathop{\sum }\nolimits }_{n=1}^{l}1/{p}_{n}$
5.3:	let, *W*_*i*_ = *W*_*i*_/*l*
6.0:	For each Weight *W*_*i*_
6.1:	Compute normalized weight as: ${W}_{i}^{normalized}=Wi/{\mathop{\sum }\nolimits }_{i=1}^{l}(Wi)$
6.2:	Compute $NS{A}_{i}=F\times {S}_{w}\times {W}_{i}^{normalized}$
	*NSA*_*i*_ is the number of word images to be augmented for each unique words in the lexicon.

### Generative adversarial networks (GANs)

Using successful generative modeling, a more domain-specific alternative to data augmentation can be found. Even though this is not well understood, data augmentation is a simpler version of generative modeling. Generative modeling offers a way to increase the number of training samples in challenging domains or domains with limited data. Deep reinforcement learning, for example, has seen a lot of success with GANs for Latin script (Arjovsky & Bottou, 2017).

When we generate new examples from an existing data set, we’re using generative modeling, which is an unsupervised machine learning task in which regularities or patterns in the input data are automatically discovered. When it comes to developing generational models, GANs shows promising results. They recognize the problem as one requiring supervised learning using two models: the generator and the discriminator. The generator model learns to generate new examples, while the discriminator model determines whether or not the new examples are real or fake (generated). GANs are an exciting and rapidly evolving field that fulfills the promise of generative models to provide proper examples in a wide range of problem areas, particularly image-to-image translation tasks (Goodfellow et al., 2020).

**Figure 2:**
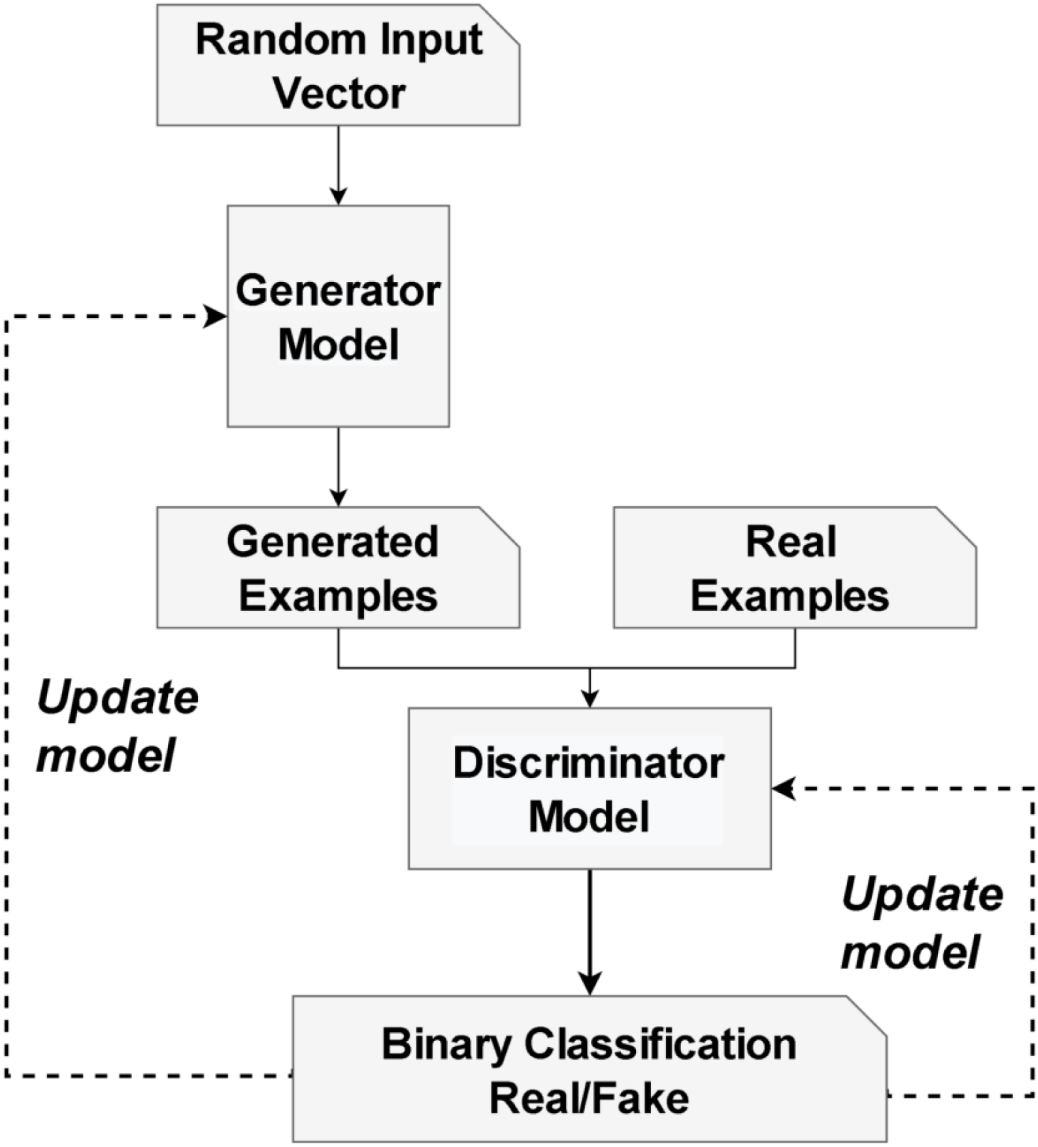
The architecture of a generative adversarial network (GAN). The illustration is adapted from Goodfellow, Bengio & Courville (2016).

The GAN architecture was first described by Goodfellow et al. (2014). A basic GAN consists of two networks: a generator (*G*) and a discriminator(*D*). The *G* network generates data with a structure identical to training data while the *D* network attempts to classify the observations as “real” or “generated”. Each time a new sample is generated, the generator sends a new batch to the discriminator, together with actual examples from the domain. The discriminator is updated in the subsequent round to improve its ability to distinguish between real and fake samples, and the generator is updated according to the degree to which the generated samples deceived the discriminator. Figure 2 illustrates an example of a model architecture for a Generative Adversarial Network (GAN). The generator makes an attempt to convince the classifier that the samples it generates are authentic. Once the generator reaches convergence, the samples generated by it become indistinguishable from real data. Table 3 summarizes the most significant findings from the two GAN sub-networks.

**Table 3:** Input and output of the generator and discriminator.

	Generator (*G*)
Input	A vector of random numbers
Output	False examples that attempt to be as persuasive as possible
Goal	Create fictitious data that is indistinguishable from the training dataset’s members
	**Discriminator** (*D*)
Input	1- Actual examples from the training set
	2- False examples derived from the Generator
Output	Probability that the input example is genuine
Goal	Distinguish genuine examples from those generated by the Generator.
	and the real examples coming from the training dataset

Recently, Jha & Cecotti (2020) have presented a GAN architecture based on Convolutional Neural Network (CNN). This network is very similar to the one presented by Goodfellow et al. (2014) with a modification to the Generator *G* and Discriminator *D* networks to generate images of handwritten words particularly. The architecture of the generator and discriminator was rearranged to provide a significant increase in accuracy. It takes as an input image of size *N*_*x*_ × *N*_*y*_ × *N*_*z*_, where *N*_*x*_ × *N*_*y*_ represents the dimensions of the image, and *N*_*z*_ represents the number of channels, *i.e.,* the color space. In our case here *N*_*z*_ = 1 since we are dealing with grayscale images. At the output layer of the generator the sigmoid function was used and the loss function was chosen to be binary cross-entropy. The discriminator layers in Jha & Cecotti (2020) was arranged as follow:

• Layer.01: Input Layer (image).
• Layer.02: Hidden layer 1: Convolution Layer.
• Layer.03: Hidden layer 2: Convolution Layer.
• Layer.04: Hidden layer 3: Fully Connected Layer.
• Layer.05: Hidden layer 4: Convolution Layer.
• Layer.06: Output Layer: Fully Connected Layer.

The generator layers are based on a deconvolutional neural network with three deconvolutional layers along with interpolation and it is arranged as follow:

• Layer.01: Input Layer (Noise Vector Input).
• Layer.02: Hidden layer 1: Deconvolution Layer.
• Layer.03: Hidden layer 2: Deconvolution Layer.
• Layer.04: Hidden layer 3: Deconvolution Layer.

**Figure 3:**
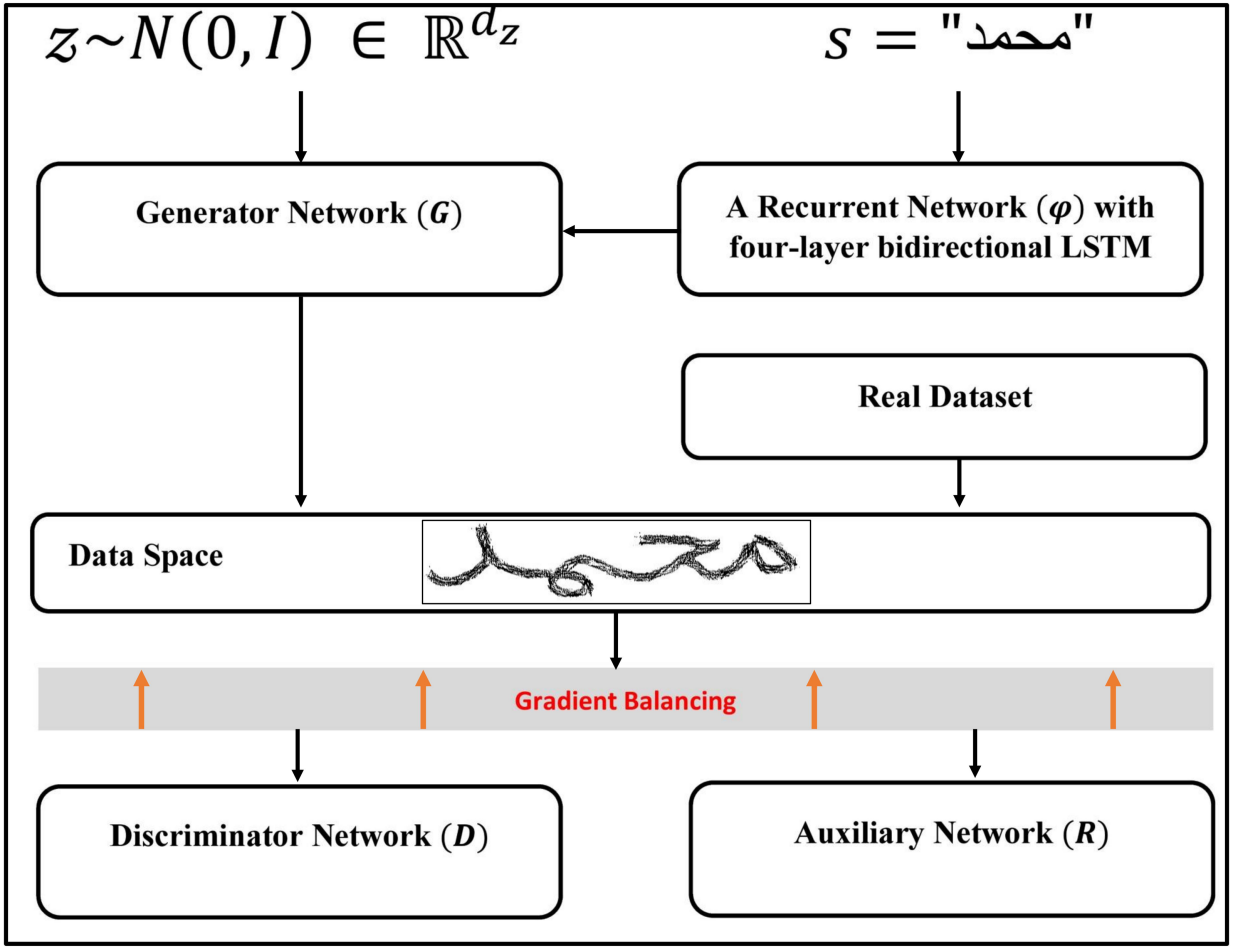
The GAN model’s architecture, as presented by Alonso, Moysset & Messina (2019). The illustration is adapted from Alonso, Moysset & Messina (2019).

On the other hand, Alonso, Moysset & Messina (2019) proposed an adversarial architecture similar to Goodfellow et al. (2014), with the addition of bidirectional LSTM recurrent layers to encode the character sequence to be produced. In order to control the textual content of the generated images, they have also introduced an auxiliary network for text recognition. They obtained an embedding of the rendered word using bidirectional LSTM recurrent layers and then passed it to the generator network. The main idea was to embed each word into a 128-dimensional feature, which is then fed into a BigGAN (Brock, Donahue & Simonyan, 2018) network architecture. The textual content of the generated images was controlled using an auxiliary classifier (Odena, Olah & Shlens, 2017). The gradients stemming from *R* and *D* are balanced (to account for their different magnitude) and finally back-propagated to *G*. The full Architecture of the adopted GAN network in this paper can be described in Fig. 3. *D*, *R*, *G*, and *φ* are specifically trained to minimize the following objectives (Alonso, Moysset & Messina, 2019):

(1) \begin{eqnarray*}{L}_{D}= & -{\mathbb{E}}_{(x,s)\sim {p}_{\text{dat}}}[\min \nolimits (0,-1+D(\mathbi{x}))] & -{\mathbb{E}}_{\mathbf{z}\sim {p}_{\mathbi{z}}\mathbi{s}\sim {p}_{\mathrm{w}}}[\min \nolimits (0,-1-D(G(\mathbi{z},\varphi (\mathbi{s}))))]{L}_{R}= & +{\mathbb{E}}_{(x,\mathbi{s})\sim {p}_{\text{dat}}}[\mathrm{CTC}(\mathbi{s},R(\mathbi{x}))]{L}_{(G,\varphi )}= & -{\mathbb{E}}_{\mathbf{z}\sim {p}_{z}\mathbi{s}\sim {p}_{\mathrm{w}}}[D(G(\mathbi{z},\varphi (\mathbi{s})))] & +{\mathbb{E}}_{\mathbf{z}\sim {p}_{n},\mathbi{s}\sim {p}_{\mathrm{u}}}[\mathrm{CTC}(s,R(G(\mathbi{z},\varphi (\mathbi{s}))))]\end{eqnarray*}

where, *p*_*dat*_ the joint distribution of real [image, word] pairs, *p*_*z*_ a prior distribution on input noise and *p*_*w*_ a prior distribution of words which may differ from the actual word distribution in the dataset.

The approach follows the GAN paradigm (Goodfellow et al., 2014), where the resulting image is evaluated by a text recognition network *R* in addition to the discriminator *D*. While *D* encourages the creation of realistic-looking handwriting, *R* encourages the creation of readable text that is true to the input text.

**Figure 4:**
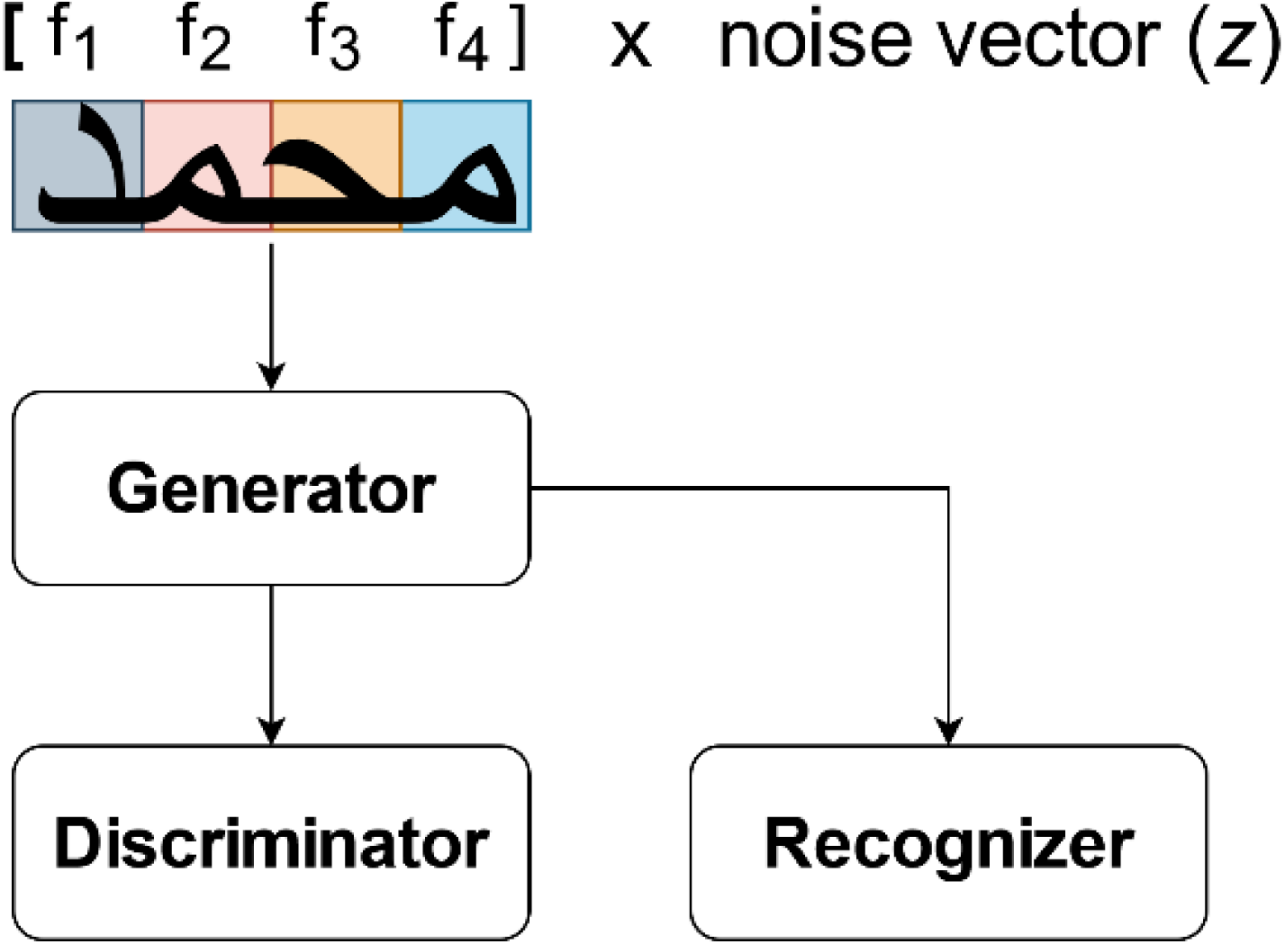
The ScrabbleGAN model’s architecture, as presented by Fogel et al. (2020).

Rather than generating the image from an entire word representation, as in Goodfellow et al. (2014), Alonso, Moysset & Messina (2019), Jha & Cecotti (2020), each character is generated independently, utilizing CNN’s overlapping receptive field property to account for the influence of neighboring letters in ScrabbleGan which presented by Fogel et al. (2020). ScrabbleGAN builds on the architecture of the previous networks, but with enhancements to produce more realistic word images. This is because handwriting is a highly localized process in which each letter is influenced solely by the letters preceding and following it. This network follows the previous GAN paradigms, but with a change to the generator network, which is the method’s key technical novelty. As a result, *G* is a concatenation of identical class conditional generators (true/false classifiers). While the discriminator *D* encourages the use of realistic images, the recognizer *R* encourages the use of readable text by effectively distinguishing gibberish from real text. This architecture seeks to minimize the sum of the two networks’ loss terms *l*. Figure 4 shows the architecture overview for the case of generating the word 
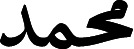
 . For each character, a filter *f*_∗_ is selected from a filter-bank *ς* that is as large as the alphabet, for example *ς* = *f*_*Alif*_, *f*_*Baa*_, …, *f*_*Yaa*_. Four-character filters are concatenated *f*_1_, *f*_2_, *f*_3_, *f*_4_ multiplied by the noise vector *z* and fed into the generator *G*. Both the discriminator (*D*) and the recognizer (*R*) use the resulting image, ensuring that the style and data fidelity are both upheld.

(2) \begin{eqnarray*}l={l}_{D}+\lambda .{l}_{R}\end{eqnarray*}

where, *l*_*D*_ and *l*_*R*_ are the loss term of *D* and *R* respectively. Also, it has been found by the authors Fogel et al. (2020) that better *R* does not lead to better general performance (generation). This is due to the LSTM layers in the RNN network learning an implicit language model and thus being able to identify the correct character even when it is not clearly written. As a result, the recurrent head is removed to force *R* to make decisions solely on visual features.

## A two-stage generative adversarial network based adaptive data augmentation technique

In this section, we present our two-stage GAN-based adaptaive data augmentation technique. The discriminator network makes an attempt to differentiate between the samples taken from the training data and the samples taken from the generator. The discriminator’s initial training data is on a known dataset. It is trained by feeding it samples from the training dataset until it achieves a satisfactory level of accuracy. The issue arises when the training data is skewed, causing the discriminator network to take examples from an imbalanced domain as input. As a result, during training, the discriminator will use some instances as positive examples more than others. Since this issue was not addressed in Alonso, Moysset & Messina (2019), Fogel et al. (2020), we present a two-stage technique to train the network which is based on ScrabbleGan as proposed by Fogel et al. (2020).

When the generated samples from the minority are fed into the discriminator, the training samples are typically classified as fake samples. It’s because the discriminator can’t find the corresponding class with fewer samples. Second, if the generator tries to fool the discriminator by generating realistic samples, it usually focuses on the majority class to optimize its loss function, causing the model to fail on the minority classes. A final issue is that the network tends to skew the distribution of the majority class space, leading to a slight over-fitting of the results. There must be enough data from both the class components for GANs to be useful, and they must also define an optimal distribution to address these problems.

Our proposed method addresses these issues by employing both majority and minority samples in adversarial training. To accomplish this, we must first balance the training data using our adaptive data augmentation algorithm, which we introduced previously in *“Adaptive Data Augmentation Algorithm”* subsection.

Following that, we will use this balanced data to train the adversarial network which is proposed by Fogel et al. (2020). This network follows the GAN paradigms presented by Goodfellow et al. (2014); Alonso, Moysset & Messina (2019); Jha & Cecotti (2020). The generator network *G* is the main technical innovation of this method. The detailed process of this network were presented in *”Generative Adversarial Networks (GANs)”* subsection. Unlike Fogel et al. (2020), which allows for varying word and image lengths, the generator network in Goodfellow et al. (2014); Alonso, Moysset & Messina (2019); Jha & Cecotti (2020) can only generate images with a fixed width across all word lengths.

**Figure 5:**
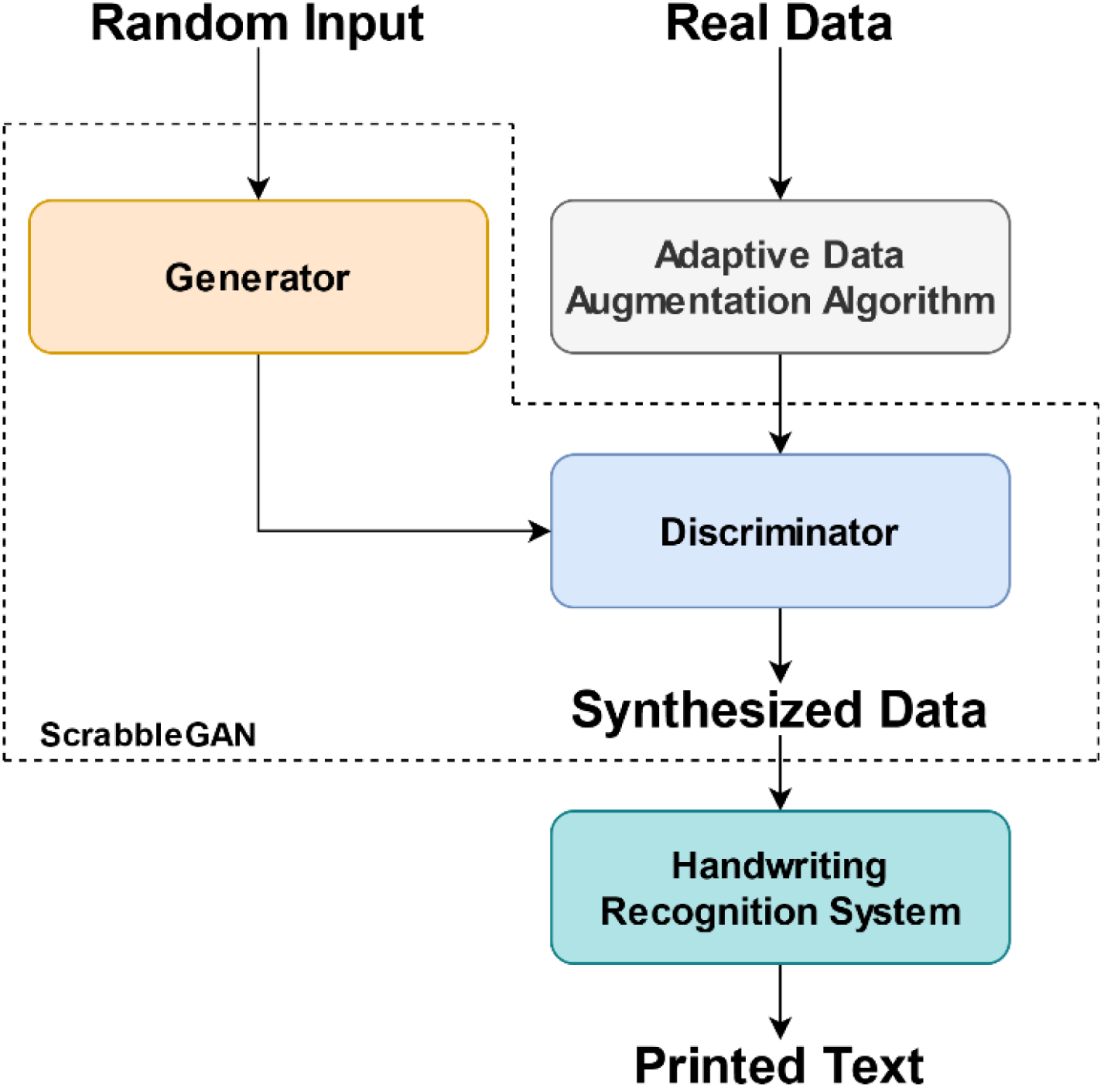
The two-stage Arabic handwriting synthesizer proposed.

Our idea is schematically presented in Fig. 5. Even though our model learns information from both the majority and the minority classes, the goal is to generate more samples for the minority classes to enhance the accuracy rate of the handwriting recognition system.

## Experimentation and results

The following subsections present the IFN/ENIT and AHDB datasets, the process of developing a synthesizer for an Arabic handwritten text, the evaluation results, and a comparison to state-of-the-art systems.

### IFN/ENIT dataset

**Figure 6:**
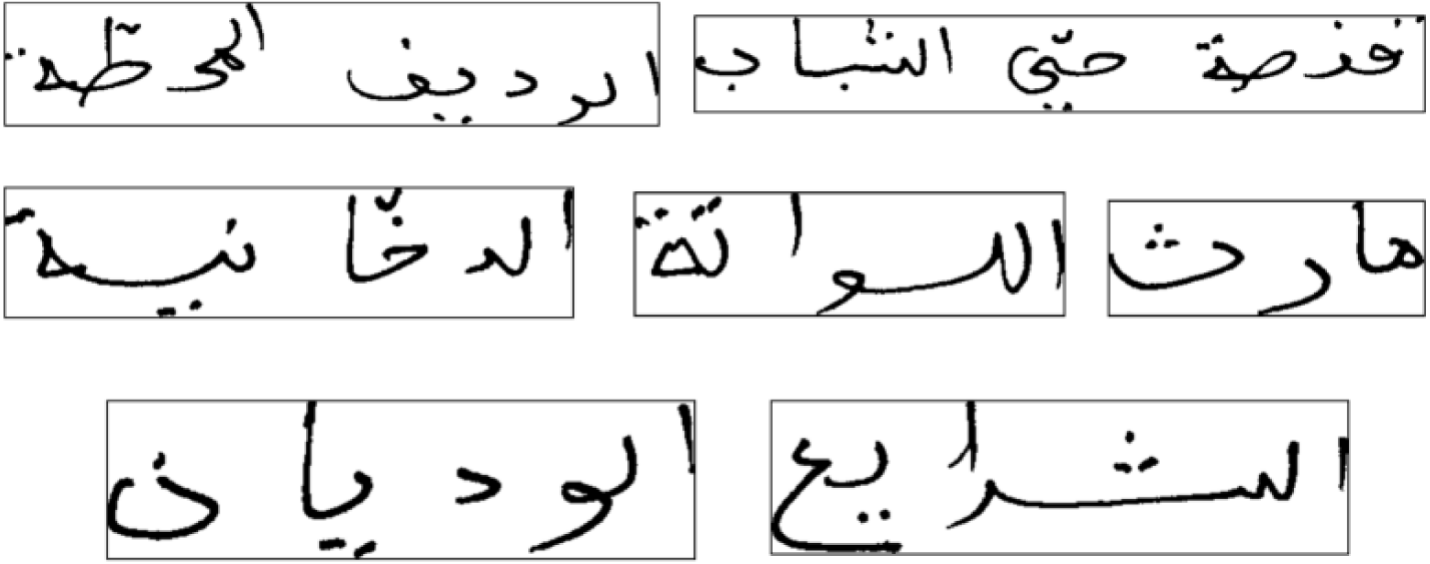
Samples from the IFN/ENIT dataset.

For handwritten Arabic text recognition research, the IFN/ENIT dataset is the most widely used and popular dataset published by Pechwitz et al. (2002). It contains over 27,000 handwritten Arabic names for Tunisian cities. The lexicon includes 937 place names. A Ground Truth (GT) file has been compiled for each word in the dataset. This file contains information about the word, such as its baseline position and the specific characters used. Figure 6 depicts image samples from the IFN/ENIT dataset.

### AHDB dataset

**Figure 7:**
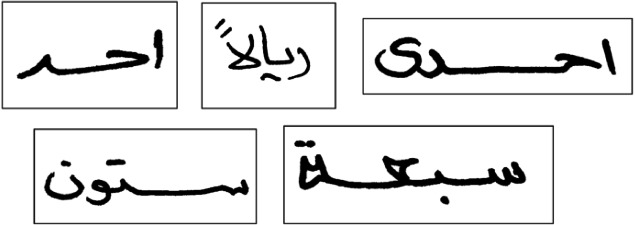
Samples from the AHDB dataset.

The Arabic Handwritten dataset (AHDB), created by Al-Ma’adeed, Elliman & Higgins (2002), consists of approximately 10,081 handwritten Arabic words representing cheque numbers and quantities, as well as the most commonly used words in Arabic script The lexicon contains 96 words, 67 of which are handwritten words that directly relate to handwritten cheque numbers. The remaining 29 words were commonly used Arabic words. Figure 7 depicts several images from the AHDB dataset as an example.

**Figure 8:**
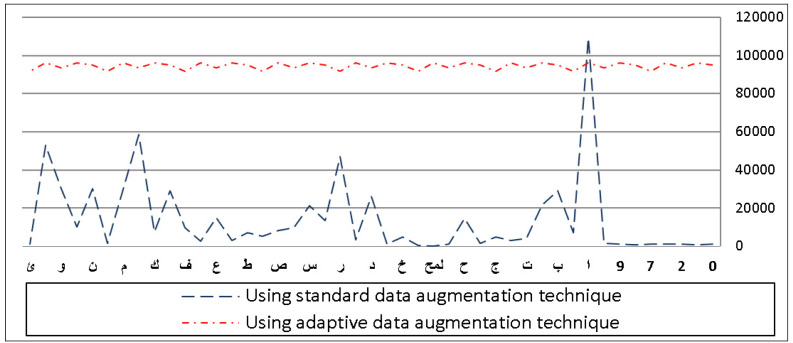
Frequency distribution of characters before and after applying adaptive data augmentation on the IFN/ENIT dataset.

### Applying adaptive data augmentation algorithm

Using our adaptive data augmentation algorithm, Fig. 8 shows the frequency distribution of the IFN/ENIT characters compared to the standard algorithm. As we have seen, increasing the number of minorities helps us achieve class balance. We can see that there is a significant difference in the appearance of certain characters in the dataset prior to using our method. For example, the letter Alif 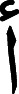 is the most occurring class, compared to the other letters which are significantly few in numbers in the dataset.

**Figure 9:**
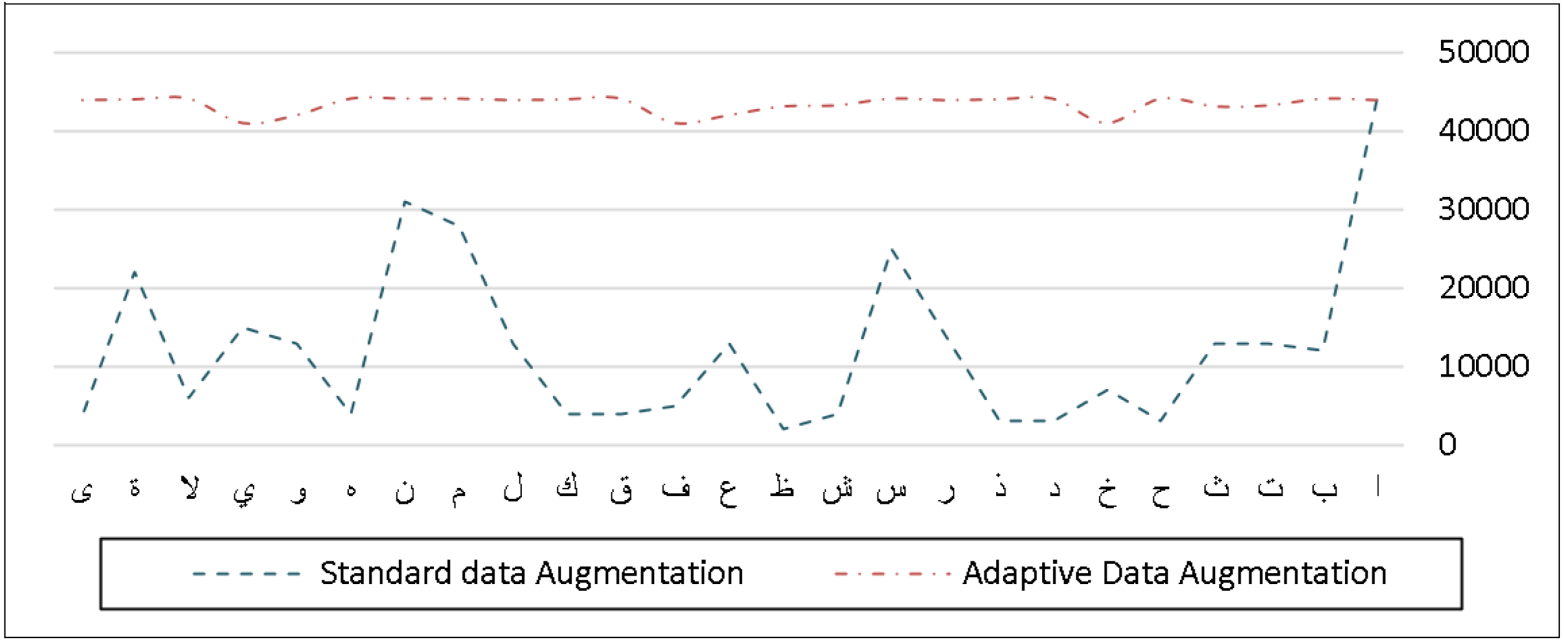
Frequency distribution of characters before and after applying adaptive data augmentation on the AHDB dataset.

The AHDB dataset, on the other hand, has about 10,000 handwritten Arabic words and 31,200 characters that can be utilized for the most frequent Arabic and handwritten cheques. Unlike the IFN/ENIT dataset, this one does not contain a significant number of Arabic characters because it was designed to identify the most often used Arabic words and terminology in cheque writing. In comparison to the original distribution of the characters, Fig. 9 shows the frequency distribution of the characters after our adaptive data augmentation approach is performed.

### Handwritten arabic words synthesizer

We have used the GAN architecture for the adversarial generation of handwritten Arabic text images, which is forked from Fogel et al. (2020). The full implementation of this network is publicly available at GitHub (Krber, 2020). Each image was resized to a fixed height of 32 pixels while retaining the original image’s aspect ratio.

We have used the standard metrics for GAN performance evaluation, namely Fréchet Inception Distance (FID) (Heusel et al., 2017) and Geometry Score(GS) (Khrulkov & Oseledets, 2018). The FID calculates the distance between feature vectors calculated for real and generated images. The FID score is used to assess the quality of images generated by generative adversarial networks, and it has been demonstrated that lower scores are associated with higher-quality images. GS, on the other hand, is assessing the quality of the generated samples and detecting various levels of mode collapse.

We have trained the model shown in Fig. 5 twice on the IFN/ENIT and AHDB datasets, respectively. First, it was trained directly on the original datasets without using our adaptive augmentation algorithm. The datasets were then balanced in the second time using our adaptive data augmentation described previously, and the balance datasets were used to train the our word-synthesizer network.

**Figure 10:**
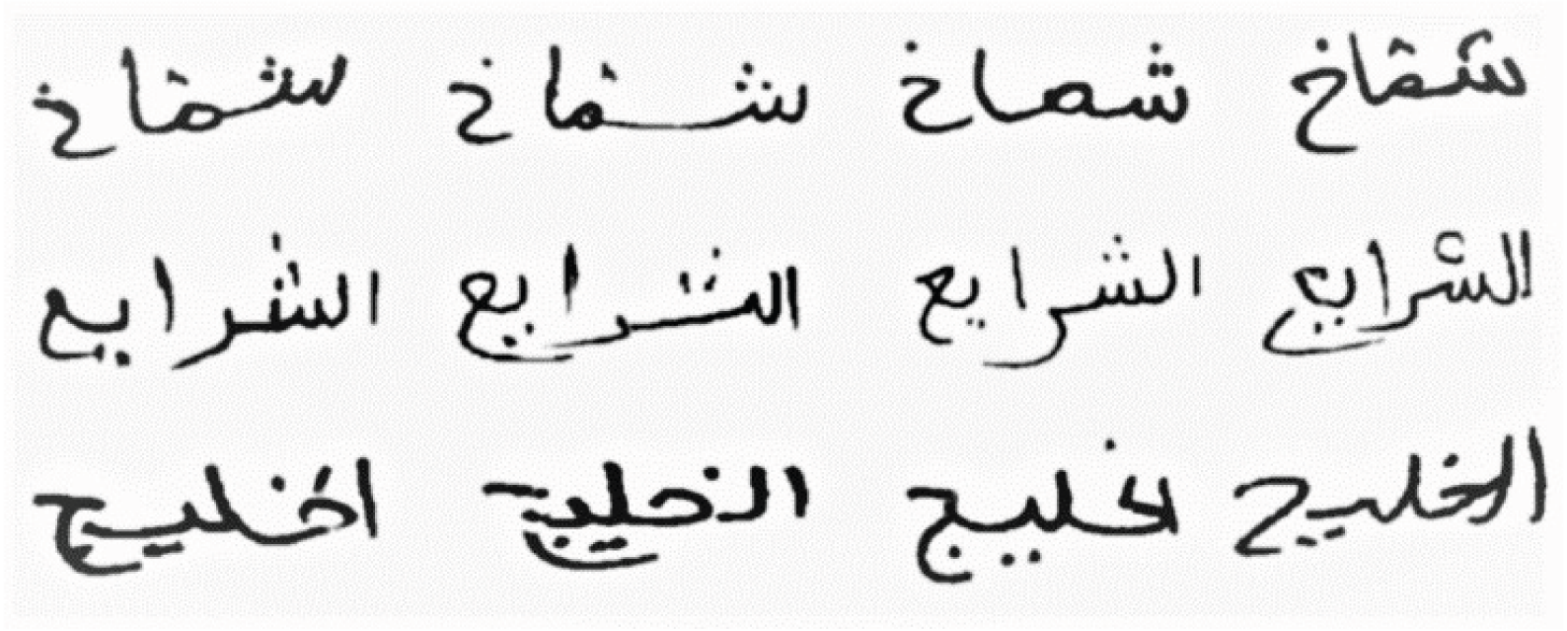
IFN/ENIT handwritten Arabic text synthesized by ScrabbleGAN. For each column, a single noise vector was used, while for each row, a different noise vector was used.

**Figure 11:**
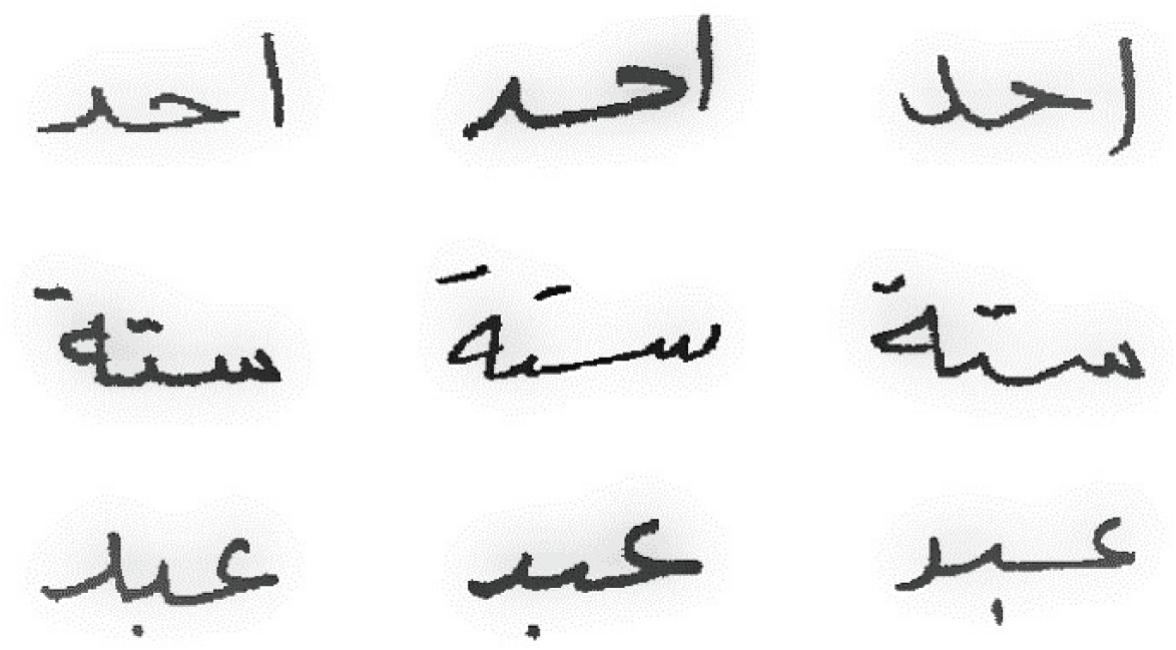
AHDB handwritten Arabic text synthesized by ScrabbleGAN. Each column was generated using the same noise vector while each row was generated by a different noise vector.

The training loop starts with the generator receiving a set of random seeds and transcriptions from a random word list. For this part, we’ve chosen random words from the dataset and their transcriptions as input to produce a set of fake images. We changed the noise vector *z* that was fed into the network to generate different handwriting styles. Figure 10 shows some examples of synthesized words from the IFN/ENIT dataset generated in different handwriting styles. Every 10, 000 iterations, we computed the FID (with 10, 000 real and 10, 000 synthesized images) and the GS (with 5, 000 real and 5, 000 synthesized images, 100 repetitions, and default settings for the other parameters). Then we have selected the best FID and best GS among the various runs independently. We have to depend on visual inspection to review the textual content. We obtain the best FID of 38.79 and GS of 18.87 × 10^−3^ , while the synthesized image is both readable and realistic. Next, we have retrained the same network but this time on the AHDB dataset after it was balanced using our adaptive data augmentation algorithm. Figure 11 shows some examples of synthesized words from the AHDB dataset generated in different handwriting styles.

As before, we computed the FID (using 5,000 real and 5,000 synthesized images) and GS (using 2,000 real and 2,000 synthesized images, 100 repetitions, and default settings for the other parameters) every 2,500 iterations. Then, independently of one another, we determined the best FID and GS for each run. We have to depend on visual inspection to review the textual content. The best FID and GS are 27.70 and 11.40 × 10^−3^, respectively, with a readable and realistic image generated. Compared to the IFN/ENIT, the visual quality of the synthesized images is much better which are expected since the AHDB dataset is much simpler in term of the word content than the IFN/ENIT dataset.

### Handwritten arabic word recognition

In this section, the value of the data generated for handwritten text recognition will be evaluated. The Recurrent Neural Network Library (RNNLIB) (Graves, 2016) was utilized in the development of the experimental models. There are many different deep learning architectures included in this library, including: (LSTM, BiLSTM, and RNN). Connectionist Temporal Classification (CTC) (Graves et al., 2006) is also included, allowing the system to transcribing sequence data that has not been segmented.

In each of the forward and backward layers, our network contains 200 extended LSTM memory blocks. There is one memory cell in each memory block, as well as an input gate, an output gate, a forget gate, and three peepholes. *tanh* is used for the cell input and output activation functions, while logistic sigmoid is used for the activation of the gate. There has been a decaying learning rate used in all CTC systems. Initially, the rate of learning was set at 0.001 and gradually decreased to 0.0001.

#### Training the classifier using GAN generated text only

In this part, we investigate the use of synthesized text as training data for the classifier. We wanted to know if a classifier trained on generated text would be capable of recognizing handwritten text. For the experimentation, we use the Arabic character’s shapes as modeling units (Ahmad & Fink, 2016). We ended up with a total of 157 models in our recognition system. To train our recognizer, we use generated text with three different writing styles in the first set of experiments.

We use the IFN/ENIT lexicon in all of its variations to generate text. We generated 20K random images for each style, which corresponded to 937 words in the lexicon, *i.e.,* each word in the lexicon has about 21 samples. As a result, the total number of samples produced is 60K which is used to train our deep learning recognition network. After training the recognizer, we tested it by recognizing word images from set *d*, *e* and *f* of the IFN/ENIT dataset.

Our next experiment involved training another recognizer on generated text from the AHDB dataset. We generated 5000 random images for each style in this experiment, which corresponded to 96 words in the lexicon. That is, each word in the lexicon has approximately 52 samples. As a result, we generated 15K samples, which we used to train our deep learning recognition network. We tested the recognizer after training it by recognizing word images from the original AHDB dataset. The evaluation results of these two experiments are presented in Table 4.

**Table 4:** Word accuracy rate for IFN/ENIT and AHDB datasets using only the generated texts as training data.

Dataset	No. of styles/sample	Test set	No of generated images	WAR%
		*d*		78.16%
IFN/ENIT	3	*e*	60,000	61.09%
		*f*		50.38%
AHDB	3	–	15,000	85.16%

As shown in the table, the recognition rate is acceptable when the recognizer is trained exclusively on generated text and not on the original data. In the next section, we will see the performance of the recognizer when we train it with generated text and original data together.

#### Training the classifier using the original and the generated texts combined

In this section, we will consider inserting the generated text into the original dataset. We intend to assess the benefits of data augmentation using GAN to improve the recognition rate even further. So, in this experiment, we trained two more recognizers from scratch that were identical to the recognizer presented in the previous experiment. The evaluation results of these two experiments are presented in Table 5. As shown in the table, significant improvements are obtained when training the recognizer with generated text and original data. On set *d* it leads to improvement in recognition rate by 5.96%. On set *e* we can see that the recognition rate has improved by 5.73% while on set *f* the improvement of accuracy rate was increased from 87.94% to 93.77%.

**Table 5:** Word accuracy rate for IFN/ENIT and AHDB dataset using the original datasets and the expanded datasets with the generated texts as training data.

Dataset	Train-test configuration	Original dataset	Expanded dataset
	*abc* − *d*	93.19%	97.15%
IFN/ENIT	*abcd* − *e*	90.14%	95.87%
	*abcde* − *f*	87.94%	93.77%
AHDB	70% training, 30% testing	96.0%	99.30%

#### Comparative analysis with other State-of-the-Art Systems

To demonstrate the proposed method’s performance, we compare our system to state-of-the-art systems evaluated on the IFN/ENIT and AHDB datasets. Table 6 compares the recognition accuracy of the most up-to-date systems using the IFN/ENIT and AHDB datasets. Numerous researches on the recognition of handwritten Arabic text using the IFN/ENIT and AHDB datasets have been conducted. Although we did not set out to achieve the lowest error rate in this paper, we did want to measure the improvement in accuracy rate when adding synthetic images to the original data sets, and our system still achieves among the highest accuracy rates.

**Table 6:** Comparative evaluation of other state-of-the-art systems using the IFN/ENIT and AHDB datasets.

	Train–test configuration			AHDB dataset
Reference system	*abc* − *d*	*abcd* − *e*	*abcde* − *f*	–
Abandah, Jamour & Qaralleh (2014)	98.96%	93.46%	92.46%	–
Ahmad, Fink & Mahmoud (2014)	98.08%	94.93%	92.30%	–
Ahmad & Fink (2015a)	97.22%	94.76%	93.32%	–
Elleuch, Tagougui & Kherallah (2015)	83.70%	–	–	–
Ahmad and Fink (2019)	97.71%	94.76%	93.32%	–
Ghanim, Khalil & Abbas (2020)	99.00%	95.60%	–	–
Hassan, Mahdi & Mohammed (2019)	–	–	–	99.08%
Eltay, Zidouri & Ahmad (2020)	98.99%	95.05%	93.07%	98.10%
Zafar & Iqbal (2020)	–	–	–	97.80%
Present work	97.15%	95.87%	93.77%	99.30%

## Conclusion

Handwritten text recognition is an exciting and challenging research area. Having sufficient labeled data to train a recognizer is one of the requirements for good performance. In this paper, we have evaluated a generative-adversarial model for producing handwritten images of synthetic words. We have also described our adaptive data augmentation algorithm and how we can combine it with this GAN model to improve the recognition performance of deep networks on handwritten text recognition tasks. We observe from the results that this model can generate realistic word images in Arabic. In one of the approaches, we investigated how well a recognizer performs when it is trained only with synthesized text generated using different styles. The results were promising considering the recognizer learns from the generated text only without looking at the original data. Using a recognizer trained on the generated text and original dataset performed significantly better than the recognizer trained on generated text only. As a future work, we can consider the problem of generating full-text lines instead of words. Also, modifying the architecture of the generator to further enhance the Fréchet Inception Distance (FID) and Geometry Score (GS) is an interesting future work.

## Supplemental Information

10.7717/peerj-cs.861/supp-1 Supplemental Information 1 Adaptive data augmentation code

10.7717/peerj-cs.861/supp-2 Supplemental Information 2 Adaptive data augmentation and ScrabbleGAN

## Additional Information and Declarations

### Data Availability

The following information was supplied regarding data availability: The data is available at GitHub and Sourceforge: - https://github.com/Nikolai10/scrabble-gan
- https://sourceforge.net/projects/rnnl/.
